# The importance of adverse childhood experiences for labour market trajectories over the life course: a longitudinal study

**DOI:** 10.1186/s12889-021-12060-5

**Published:** 2021-11-08

**Authors:** Claus D. Hansen, Mette J. Kirkeby, Kristian G. Kjelmann, Johan H. Andersen, Rasmus J. Møberg

**Affiliations:** 1 Department of Sociology and Social Work, Aalborg University, Fibigerstræde 13, 63, DK-9220 Aalborg, Denmark; 2 Department of Clinical Medicine, Danish Center for Clinical Health Service Research (DACS), Aalborg University, Aalborg, Denmark; 3 Department of Occupational Medicine, Danish Ramazzini Centre, University Clinic, Herning, Denmark

**Keywords:** Negative life events, Disability pension, Administrative data, Sequence analysis, School-to-work transition

## Abstract

**Background:**

Transitioning from school to work is important in influencing people’s trajectories throughout their life course. This study investigated the extent to which adverse childhood experiences (ACEs) were associated with differences in labour market trajectories for young adults in the context of a Nordic child care regime with low levels of child poverty.

**Methods:**

Information on labour market participation, educational events, and public transfer records was recoded into seven state spaces for each month between ages 16 and 32 for a cohort of Danish adolescents born in a rural county in 1983 (*N* = 3373). Cluster analysis of the sequences using the optimal matching algorithm was used to identify groups with similar trajectories. Multinomial regression was used to assess the association between self-reported ACEs and cluster membership, taking gender and family of origin into account.

**Results:**

‘In employment’ was the state space in which the young adults spent the most time over their early life courses (mean: 85 out of 204 months; 42%). Cluster analysis identified three clusters. Cluster 3 was most distinct, where the mean time ‘outside the labour market’ was 149 months (73%), and only 17 months (8%) were spent ‘in employment’. Cumulative ACEs increased the probability of being included in Cluster 3 (OR: 1.51). Experiencing parental divorce (OR: 3.05), witnessing a violent event (OR: 3.70), and being abused (OR: 5.64) were most strongly associated with Cluster 3 membership.

**Conclusions:**

Labour market trajectories among adolescents with a higher number of ACEs consisted of more time outside the labour market, compared to adolescents who had experienced fewer adversities. The lasting consequences of childhood adversity should be taken more into account in welfare policies, even in countries such as Denmark, with high social security levels and high-quality universal childcare.

**Supplementary Information:**

The online version contains supplementary material available at 10.1186/s12889-021-12060-5.

## Background

Adverse childhood experiences (ACE) are gaining attention as a component in explaining negative life course outcomes [1]. Research has documented the effect of ACEs on physical [2] and mental health-related outcomes [3–6]. The cumulative effect of ACEs is increasingly being researched [5], and knowledge of the different outcomes of specific ACE combinations is being revealed. Children exposed to maltreatment have an increased likelihood of experiencing other ACEs and can consequently be considered particularly vulnerable [7]. Other studies have demonstrated that ACEs are associated with a higher risk of smoking [6, 8, 9], alcohol consumption [5], substance abuse [10], and suicide attempts [6]. Exposure to ACEs is also linked to alcohol consumption during pregnancy [11], with possible negative outcomes for the child in utero. Research presents a convincing argument for the importance of taking adverse experiences in early childhood and adolescence into account when discussing health outcomes during people’s life courses, even if the specific mechanisms responsible for these associations are still uncertain.

### ACEs and labour market trajectories

The effect of ACEs on labour market trajectories is not well documented. Measurements of labour market outcomes in studies addressing this issue are often based on a single time point or on accumulated unemployment within a specific calendar year or at a particular chronological age [12–18]. When a single time point or calendar year is used as the measurement of labour market participation, the results are vulnerable to period effects (e.g., due to fluctuations in the economy) or effects associated with a specific chronological age in the case of birth cohort studies. Existing research on this topic thus only ascertains the implications for overall labour market trajectories to a limited extent. In addition, the timing of the ACEs, i.e., at what chronological age the adverse conditions were experienced, are not addressed.

There is, however, evidence indicating that ACEs experienced in early adolescence (from age 12 and onwards) may have more lasting consequences, e.g., on externalising behaviour, compared to those experienced at an earlier age [19]. This may be particularly relevant when it comes to labour market outcomes, because the child is going through a developmental stage in which they are trying to make decisions about their education and career, and because of the tracking of the educational system in some countries.

Three studies also neglected to include information on parents’ socioeconomic positions (SEPs) [12, 18, 20], despite documentation in a systematic literature review that parental SEP is strongly associated with ACE prevalence [20], and therefore crucial in studying the effects of ACEs. Research has shown that lower parental SEP is linked to their children’s poorer labour market outcomes [21].

Stable labour market participation, i.e., the absence of long-term or frequent spells of unemployment, is also often seen as a central component of successful (later) adulthood. Absence of stability has been shown to produce negative health consequences [22, 23]. Similarly, a scarring effect of youth unemployment on later employment opportunities has been documented [24]. This implies that the effect of ACEs accumulates to impact later life outcomes, where unsuccessful labour participation increase the risk of negative physical and psychological health consequences and vice versa, and potentially results in a downward spiral of social mobility. Identifying how ACEs are associated with early labour market trajectories is vital for understanding people’s overall life course development.

Gender is an important factor to consider in analyses of the impact of ACEs on labour market trajectories for several reasons. First, some studies have found that women are more sensitive to ACEs and that this cannot be considered an artefact of measurement alone [25, 26]. This may in part be due to the fact that more girls than boys are exposed to sexual abuse, and this type of ACE is particularly severe [25, 27]. Gender is also important because men and women embark on quite different paths through the educational system, largely due to the segregation of the labour market in many societies [28]. This means that the transitions between primary, secondary, and tertiary education, and becoming employed will be quite different for men and women in general. In a Danish context, more women than men attend tertiary education, while men enter the labour market at an earlier age. If the possible effect of ACEs depends on the context (i.e., educational system vs. being employed), this could entail gendered differences that need to be taken into account.

### ACEs in a Danish context

A substantial part of the research on ACEs originates from the United States [29], and transferring knowledge across political and cultural contexts is not always possible. The Nordic welfare states are widely regarded as ‘women friendly’ [30], not least because of their childcare policies. The Nordic child care regime [31] means that universal, high-quality child care is highly subsidised by the state, making it affordable for everyone. As a consequence, nearly 100% of all children aged four attend kindergarten, making it possible for both parents to be active in the labour market. Approximately 80% of all women are employed, and this is one important explanation for the low rate of child poverty in Denmark (2.7% in Denmark vs. 12.4% in OECD), as well as other Nordic countries (29). Another key explanation of the low child poverty may also have to do with the social democratic welfare state regime that aims to provide financial support to families in the case of long-term illness or unemployment [31].

Denmark also has a relatively high level of out-of-home care placement compared to other Nordic countries, especially from an international perspective [32]. Historically, the ideology underlying this policy has focused on cooperation with parents and out-of-home care placement as a family-oriented intervention aimed at helping children or adolescents in the context of the family.

The consequences of this context on the study of ACEs are multiple. First, due to the high level of kindergarten participation among Danish children, we could expect adverse experiences in childhood to come to the attention of professionals and lead to some kind of intervention aimed at removing, or at least reducing, the negative consequences. This may be particularly true of ACEs related to abuse or neglect. Second, because of the welfare state regime, we could expect the negative financial consequences of unemployment, divorce, or the death of a parent to be less severe than in places without this provision, although the psychological consequences might still be the same.

Studying the association between ACEs and labour market trajectories in a Danish context is important because some of the negative consequences of ACEs are alleviated by public policies. Finding an association here would imply that consequences of ACEs are mediated by dysfunctional family relations (such as consequences for self-esteem, self-concept, etc.) rather than derived consequences due to the negative socioeconomic consequences of the events.

### Aim of the paper

This paper describes differences in transition into the labour market and trajectories between education and work from ages 16 to 32 across a rural Danish birth cohort (born in 1983). We investigated to what extent ACEs were associated with differences in these labour market trajectories after taking the young adult’s family of origin (i.e., parental SEP at age 15) into account.

## Methods

### Data

The data for this study were derived from a birth cohort study (VestLiv—the West Jutland Cohort Study) of young adults born in 1983, living in Ringkjøbing County. At the age of 19–20 (*N* = 3373), the cohort was invited to answer a postal questionnaire distributed in April 2004. A total of 1869 respondents (response rate: 55%) answered the questionnaire.

Information on labour market participation, educational events, and public transfers was derived from three population registers at Statistics Denmark: The Integrated Database for Labour Market Research [33], the Student Register [34] and the Danish Register for Evaluation of Marginalisation (DREAM) [35]. The information was linked using the unique civil registration number (CPR) given to everyone at birth or upon entry to Denmark [36]. The combined information was coded into seven distinct state spaces on a monthly basis, from ages 16 to 32 (January 1999 to December 2015). The seven state spaces were compulsory school, upper secondary, vocational training, higher education, other education, employment, and outside labour market.

The database contained information on 3328 individuals; however, to analyse the state sequences, only those with complete information for more than 2/3 of the period were retained in the analyses. This means that those who migrated in or out of Denmark and stayed outside Denmark for a prolonged period were excluded, as were those who died before age 32 (*N* = 3264).

### Outcome variable: clusters of transitions and labour market trajectories

Cluster analysis (using Ward’s distance) [37] was used on the 3194 unique sequences identified in the linked dataset to create relatively homogenous groups of young adults with similar types of transitions and overall early life labour market trajectories. First, distances between the individual sequences were computed using the optimal matching algorithm, applying the transition rates between the seven state spaces as the substitution cost for the calculation [38–40]. Second, Hubert’s C (HC) [41], Hubert’s gamma (HG) [42], weighted average silhouette width (ASWw) [43], and point biserial correlation (PBC) [44] were used to assess the quality of the partitioning. Although the quality assessed by the abovementioned criteria suggested partitioning the sequences into five to nine clusters, we retained only three for the subsequent multinomial logistic regression, since further partitioning of the clusters would primarily subdivide individuals into Clusters 1 and 2, that is, those with relatively successful transitions. Analysing nine distinct clusters would also complicate cluster interpretation, and in this type of analysis, focusing on describing distinct labour market trajectory interpretations was more important than model fit indices.

### Exposure variables: ACEs

Concerns about the validity and accuracy of ACEs reported in retrospective studies have been raised [45]; however, the main problem is the occurrence of false negatives [46] and, only rarely, false positive reporting. Other research has found good cross-time reliability in ACE reporting, with slightly better reliability for women. More reliable reporting of ACEs is associated with the specificity and unambiguity of the particular ACE (e.g., the death of a parent) [45, 46]. Drawing on official reports on situations such as child maltreatment or where ACEs had led to foster care placement could have raised the validity of the exposure variable; however, this would have meant only focusing on those very severe cases where public authorities became involved, and this might have increased the likelihood of differential misclassification if certain groups were more likely to be reported to the authorities in case of suspected child maltreatment. We used a retrospective collection of data; respondents were 20 years old when asked about ACEs until the age of 16.

ACEs were measured using seven items stemming partly from a scale developed by Newcomb, Huba, and Bentler [47] and supplemented with items from The Social Stress Indicator [48]. The items covered were similar to the standard instruments used to assess ACEs [49], although commonly assessed topics, such as parental incarceration, household mental illness, and experiences of neglect, were not part of the questions. The seven questions were as follows: ‘During the first 16 years of your life…’, 1: ‘Have your parents divorced?’ 2: ‘Have you lost any of your parents because they died?’ 3: ‘Have your mother or father been unemployed for a long period?’ 4: ‘Have any of your parents abused alcohol or drugs to an extent where it caused problems in the family?’ 5: ‘Have you been abused by someone you knew?’ 6: ‘Have you witnessed a very violent event, possibly seeing someone get killed?’ and 7: ‘Have your parents suffered a life-threatening disease or accident?’ For each experience, the respondent could answer ‘yes’ or ‘no’. A cumulative measure of ACEs was computed ranging from 0 to 7.

### Confounders: family of origin and gender

To account for the effect of family of origin on young adults’ transitions from basic school and into the labour market, we included two measures of parental SEP at the end of 1997 (age 15 for the participants): parental education (or, in case the participant was living apart from their parents, that of the household’s primary caregiver) and equivalised disposable household income. We chose this age because it reflected the age up to which the self-reported ACEs were measured. To simplify the analyses, the measure of education was dichotomised to indicate whether the parent or caregiver had any education apart from basic school. Gender was included in the analyses for two reasons: first, because of the rather large gender difference in membership in the two first clusters reflecting different educational tracks for boys and girls, and second, because earlier research has shown that girls might be more vulnerable to the effects of ACEs [26]. In the analyses, all interactions between gender and the individual ACEs, as well as parental SEP and the individual ACEs, were tested. None of the interaction terms were statistically significant.

### Statistics and handling of missing data

To handle missing information from the participants in the dataset and the possible biased results arising from non-responders, multiple imputation with 25 imputations (M = 25) was employed. Several auxiliary variables (primarily register-based information on parents) that were found to be associated with the variables in the statistical model were included in the imputation process. The results reported are those derived from the multiple imputed analyses using the MI estimation procedure in STATA version 16 [50]. For this reason, all results were reported for 3264 persons with information on sequences and cluster membership. Multinomial logistic regression modelling using ACEs and family of origin as predictors of cluster membership was used to ascertain associations between ACEs and subsequent labour market trajectories, as well as successful transitions between different monthly states from age 16 to 32. Sequence analyses and clustering techniques were prepared and analysed using the TraMineR package in R [51].

### Ethics

The study and the linking of information across registers were approved by the Danish Data Protection Agency (Study No. 2009-41-3761).

## Results

**Fig. 1:**
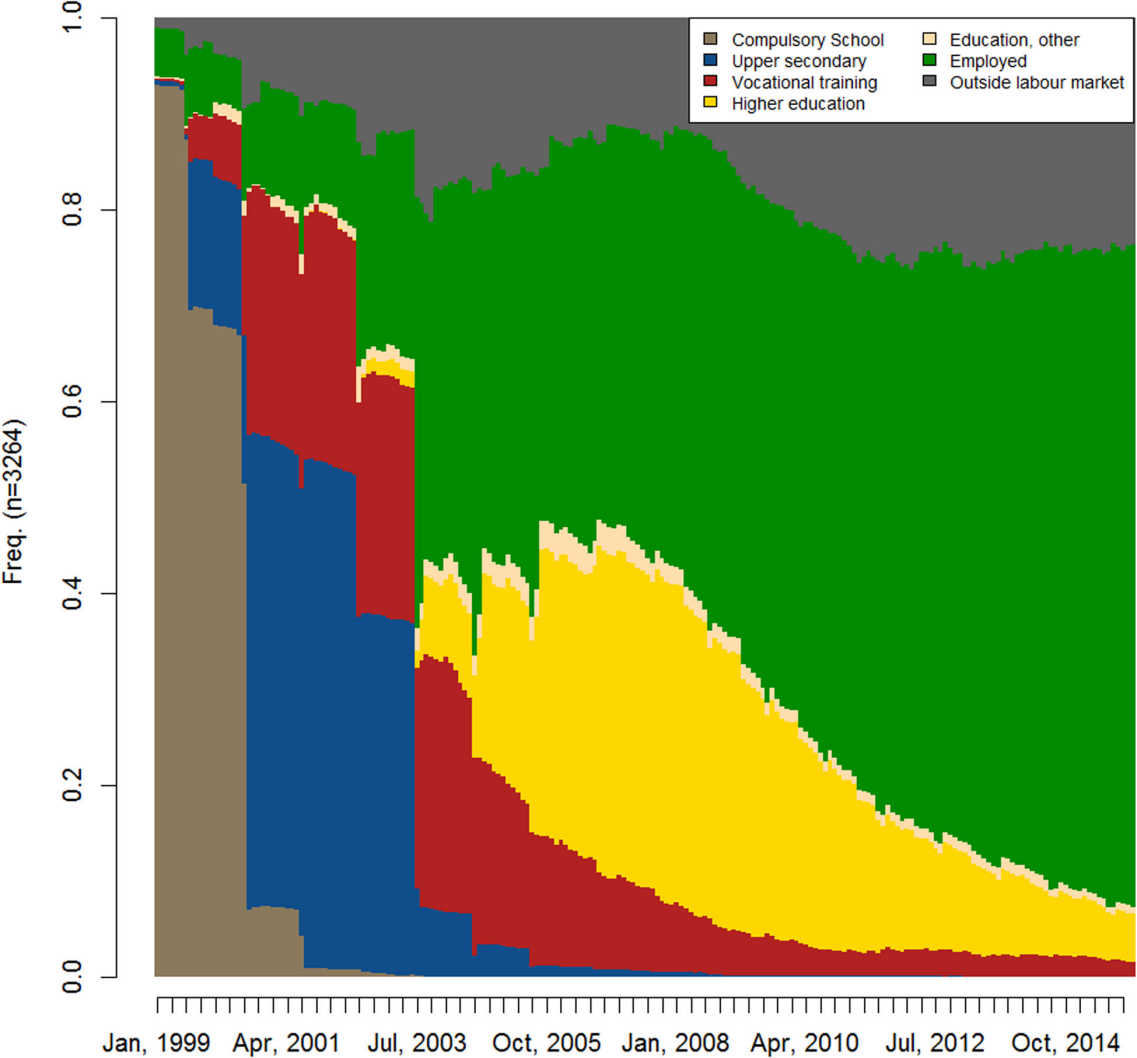
Labour market trajectories. Age 16 to 32

Figure 1 shows the state distribution plot across the 204 months of observation for the study population of 3264 individuals. The graph starts in January 1999, when the majority of the cohort still attended compulsory school. From ages 18 to 20, between 60 and 80% attained upper secondary education or vocational training. Until age 24 (Oct, 2007), nearly half of the population was still in an educational trajectory—either finishing vocational training or moving on to higher education. From age 24 onwards, most were employed, although the share of people outside the labour market increased slowly over time when looking at the distribution cross-sectionally at each time point.

**Fig. 2:**
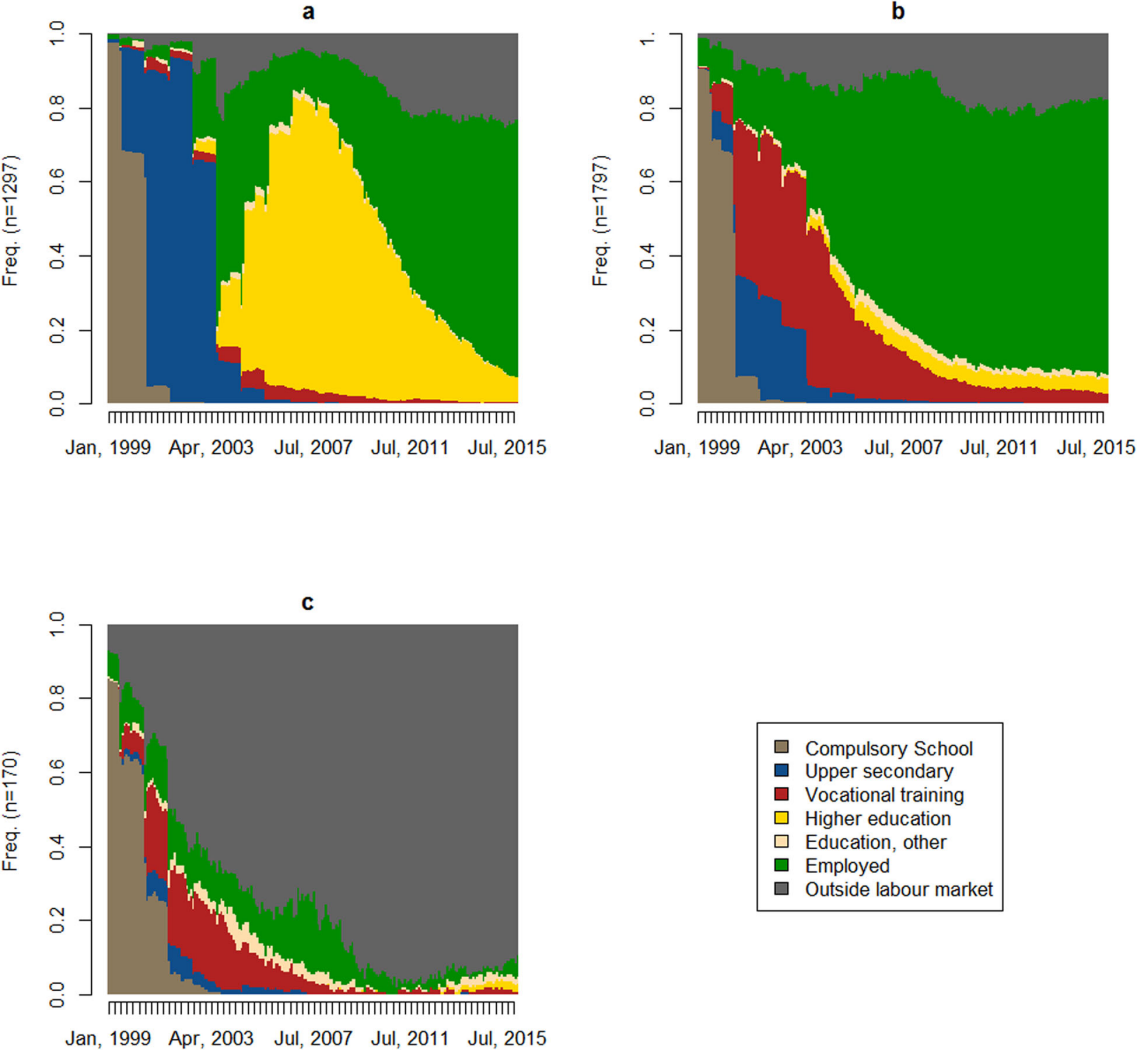
**a** Cluster 1: Trajectories with higher education. **b** Cluster 2: Trajectories with vocational training. **c** Cluster 3: Trajectories outside labour market

The overall trajectory is somewhat misleading, as can be seen from Fig. 2 a-c, where we plot the state distribution for the three clusters. In Table 1, we characterise the three clusters by gender, family of origin, and ACEs, as well as the mean duration in each state. Cluster 1 consisted of young adults who attended higher education and subsequently entered the labour market, as seen in Fig. 2a. This cluster includes a majority of women (60%) and has a family of origin with the highest average household disposable income and the lowest share of parents without secondary education. Cluster 2, in contrast, consists mostly of men who took some kind of vocational training and subsequently entered the labour market at a lower age than those in Cluster 1. This is also evident in Fig. 2b. As seen in Table 1, members of Cluster 2 had already spent nearly three-and-a-half years (42 months) more in the labour market than their peers pursuing higher education. Of this cluster, 17% had at least 1 week of sick leave after turning 30, while only 11% in Cluster 1 reported the same (results not shown). In this cluster, household disposable income was slightly lower, and the share of parents without secondary education was a little less than 50%.

**Table 1:** Sociodemographic characteristics by clusters of labour market trajectories. *N* = 3264 after multiple imputation (Percent)

	Grand Mean	Cluster 1	Cluster 2	Cluster 3
**Sociodemographic characteristics and class of origin**				
Gender (% female)	47.3	60.1	37.8	51.2
Household disposable income (mean equivalented in Dkr)	106,253	113,122	103,204	86,076
Parental education (% unskilled)	39.5	26.5	47.1	58.0
Lone parent (% living with single parent)	8.7	7.1	9.2	15.5
Number of children in household (mean)	2.4	2.4	2.4	2.1
**Mean duration of state spaces (in months)**				
Basic school	15.5	15.5	15.4	16.6
Upper secondary	19.5	33.7	11.0	2.6
Vocational training	20.8	4.1	33.5	13.3
Higher education	27.5	60.7	6.1	0.6
Other education	2.9	2.0	3.4	4.4
Employed	84.8	63.8	105.9	17.1
Outside labour market	33.0	24.3	28.7	149.4
N (%)	3264	1297 (39.7)	1797 (55.1)	170 (5.2)
**Adverse childhood experiences**				
Mean number of adverse childhood experiences	0.8	0.6	0.9	1.5
Parental divorce	20.5	15.1	22.9	35.4
Parental unemployment	18.2	14.2	19.8	32.1
Parental death	5.1	3.7	5.5	10.4
Parental drug or alcohol abuse	13.3	9.8	14.9	23.0
Abuse or neglect	5.3	3.7	5.2	18.3
Witness violent event	6.3	3.2	8.0	11.3
Parental accident or serious illness	13.0	11.7	13.3	20.4

Cluster 3 is the most distinct group, where the mean time ‘outside labour market’ amounted to 149 months, i.e., 73% of the observed time, with only one-and-a-half years (17 months, or 8% of the time) spent in the labour market. This cluster is dominated by families of origin, where nearly two-thirds of the parents had no secondary education and a significantly lower household disposable income compared to the other two clusters. As seen in Fig. 2c, when members of Cluster 3 entered their late 20s (Apr, 2010), less than 10% were in states other than ‘outside the labour market’. This is also reflected in the fact that more than 50% of the members of the cluster were on a disability pension or an equivalent, whereas only 1% reported the same in the two other clusters (results not shown).

Table 1 also shows the distribution of ACEs across the clusters. Cluster 3 had 1.5 experiences on average, while Cluster 1 averaged only 0.6. For all specific adversities, the same pattern was seen: Cluster 1 had the lowest share of members experiencing adversities, and Cluster 2 had a somewhat higher share, while Cluster 3 was markedly different. For some of the experiences in this cluster, such as abuse or witnessing a violent event, the difference was more than fourfold.

Table 2 shows the association between ACEs and cluster membership. Increasing the number of childhood adversities, regardless of the specific type, increases the probability of being a member of either Cluster 2 (OR: 1.23) or Cluster 3 (OR: 1.51), even taking gender and family of origin into account. In the bivariate analysis, all but one of the seven specific adversities (parental sickness) increased the probability of being a member of Cluster 3, and five increased membership in Cluster 2. Taking gender and family of origin into account attenuated the results, but the associations pointed in the same direction. The ACEs most strongly associated with changing membership from Cluster 1 to Cluster 3 included experiencing ‘parental divorce’ (OR: 3.05), ‘being abused’ (OR: 5.64), and ‘witnessing a violent event’ (OR: 3.70).

**Table 2:** Adverse childhood experiences (ACEs) (until age 16) and labour market trajectories (age 16–32). *N* = 3264 after multiple imputation. Multinomial logistic regression relative risk ratios (95% CI)

	Cluster Membership	Model 1 (Bivariate associations)	Model 2 (Adjusted for SES)
**Adverse childhood experiences**			
No. of adverse childhood experiences	1	1 (ref)	1 (ref)
	2	1.27 (1.16–1.39)	1.23 (1.10–1.37)
	3	1.70 (1.41–2.05)	1.51 (1.21–1.90)
Parental divorce	1	1 (ref)	1 (ref)
	2	1.67 (1.30–2.13)	1.73 (1.28–2.35)
	3	3.05 (1.69–5.50)	2.33 (1.11–4.91)
Parental death	1	1 (ref)	1 (ref)
	2	1.53 (0.96–2.45)	1.32 (0.82–2.13)
	3	2.85 (1.09–7.44)	1.99 (0.71–5.52)
Parental drug or alcohol abuse	1	1 (ref)	1 (ref)
	2	1.60 (1.21–2.11)	1.47 (1.09–1.97)
	3	2.71 (1.47–4.97)	1.95 (1.03–3.66)
Parental unemployment	1	1 (ref)	1 (ref)
	2	1.49 (1.14–1.93)	1.20 (0.91–1.59)
	3	2.82 (1.44–5.52)	1.74 (0.84–3.64)
Abuse or neglect	1	1 (ref)	1 (ref)
	2	1.43 (0.89–2.30)	1.68 (1.03–2.75)
	3	5.64 (2.41–13.22)	4.84 (1.95–12.02)
Witness violent event	1	1 (ref)	1 (ref)
	2	2.67 (1.63–4.37)	2.38 (1.45–3.90)
	3	3.70 (1.21–11.23)	2.82 (0.90–8.83)
Parental accident or serious illness	1	1 (ref)	1 (ref)
	2	1.16 (0.85–1.57)	1.05 (0.76–1.47)
	3	1.91 (0.97–3.73)	1.49 (0.73–3.04)
**Sociodemographic characteristics and class of origin**			
Gender (male vs. female)	1	1 (ref)	1 (ref)
	2	2.49 (2.14–2.88)	2.55 (2.19–2.97)
	3	1.44 (1.03–2.01)	1.56 (1.11–2.20)
Parents unskilled vs. Skilled	1	1 (ref)	1 (ref)
	2	2.47 (2.11–2.88)	2.43 (2.06–2.86)
	3	3.82 (2.66–5.49)	3.38 (2.34–4.87)
Household disposable income (in 100.000 Dkr)	1	1 (ref)	1 (ref)
	2	0.73 (0.63–0.85)	0.74 (0.63–0.87)
	3	0.29 (0.19–0.44)	0.29 (0.19–0.45)
Lone parent vs. two parents	1	1 (ref)	1 (ref)
	2	1.34 (1.01–1.76)	1.20 (0.89–1.62)
	3	2.40 (1.48–3.92)	1.60 (0.96–2.67)
Number of children in household	1	1 (ref)	1 (ref)
	2	0.91 (0.84–0.99)	0.90 (0.83–0.98)
	3	0.68 (0.55–0.83)	0.67 (0.55–0.82)

## Discussion

The results presented in this paper describe the early life course transitions between compulsory school and various educational tracks and participation in the labour market or, in more unfortunate cases, a life outside the labour market. Applying the optimal matching (OM) algorithm resulted in three relatively homogenous clusters with distinct trajectories. Cluster 3 was especially interesting, consisting of approximately 5% of the cohort, who had little success in transitioning from compulsory school into the educational system and labour market. In fact, more than half of the members of this cluster ended up on a disability pension or equivalent social transfer payment by their early 30s. This cluster came from families of origin with lower parental SEPs, and its members experienced adversities in childhood to a much higher degree than members of the other clusters. Interestingly, Cluster 3 had an equal share of men and women and very little time in either secondary or tertiary education or employment. This indicates that for men and women alike, those ending up outside the labour market may well have already been set upon that trajectory during compulsory school.

Results showed, however, that the ACEs not only predicted membership in the relatively small group that had most trouble transitioning into the labour market; they also increased the likelihood of being a member of Cluster 2, which consisted of individuals with labour market trajectories involving either vocational training or no formal/completed education beyond primary school, and involved earlier entry into the labour market than the members of Cluster 1. In Cluster 2, there was a significantly higher level of sick leave compared to the two other clusters, indicating the negative consequences of ACEs on health-related labour market outcomes. The impact of ACEs also appeared cumulative, with an increasing risk of early onset and difficulties in labour market transition with an increasing number of ACEs; this is in line with findings from similar studies [14, 15, 18].

Although earlier studies found ACEs to be more detrimental to the development of depression in women [25], our results did not indicate any heterogeneous effects of ACEs, either with respect to gender or parental SEP. Whether this reflects homogenous negative effects of ACEs across both gender and parental SEP, or has to do with the measures of ACEs used in this study, the fact that the follow-up time was only until age 32, or that labour market outcomes are less affected than symptoms of depression or other psychological characteristics, should be examined in future studies, extending the results we have found.

In this article, we focused on the association between ACEs and labour market trajectories, where the measurement of ACEs constituted indicator variables. An indication of individual changes (e.g., consequences for self-esteem and self-concept) or social environmental changes (e.g., changes in relation to parents or family constellations) could have, in turn, affected future labour market participation. It could be argued that ACEs are mediated through these changes. However, in this study, we did not have access to measures that could shed light on the mechanisms causing the differences in subsequent early adulthood labour market trajectories. As such, this study is limited to analysing ACEs as indications of these possible mechanisms.

### Strengths and limitations of the study

The strengths of this study include the long observation period, with 204 monthly observations of educational events, transfer payments, and labour market participation. This extended period not only enabled an examination of singular events, such as becoming unemployed or starting education, but it also enabled studying a sequence of transitions between distinct states that form the labour market trajectories of young adults in a Danish context in the 2000s. This approach highlights that there are groups of young people who have a hard time transitioning successfully into the labour market. ACEs increase the risk of such unsuccessful transitions, suggesting that more focus should be placed on providing help to adolescents with such experiences. Relying on register-based information on the trajectories is also a clear advantage of this study, making it possible to conduct a descriptive analysis for groups that are otherwise hard to convince to participate in surveys.

A weakness of the study is the use of retrospectively self-reported ACEs, making the results of the multinomial regression prone to bias originating in differential non-response rates across the three clusters. This possibility is suggested by the response rates across the clusters, which range from well over 60% in Cluster 1 to only 38% in Cluster 3. Despite having used multiple imputation in our paper to handle the non-response data, this procedure assumes that the mechanism producing the non-response is missing at random when taking into account the variables in the imputation model. We cannot rule out the possibility that part of the mechanism leading to differential non-response across the clusters is non-random. For this reason, the use of objective information on, for example, parental death, divorce, or extended periods of unemployment [15] could remedy some of these problems, especially if the timing of the events could be taken into account. However, for other types of ACEs, such as parental alcohol abuse, physical abuse, or violence in the family, drawing only on cases in which public authorities were involved and possibly leading to foster home care placement of the children would substantially weaken the validity of the study because of the many cases that never come to the attention of public authorities.

The measure of ACEs used in this study also did not include, for example, parental incarceration and mental health problems in the family, which are important exposures used in many studies of childhood adversity. Further, the follow-up time permitting observation only until age 32 is a weakness, as some members of clusters, especially Cluster 1, still had not completed their higher education at age 32. A drawback of the Danish context is that the population is relatively homogenous in terms of race and ethnicity. This means that social inequalities and their association with ACEs arising from the intersection of race, ethnicity, and class are not easily examined here. Conducting the study in a Danish rural context means that diversity related to race and ethnicity is very limited, making inferences to other contexts with a more mixed ethnic population difficult [52]. However, compared to the few existing studies of the association between childhood adversity and labour market participation, this study has a surplus of advantages, most notably the use of objective information on outcomes and the extended period on which it is based (i.e., 16 years).

### Directions for future studies

Future analyses of these methodological problems should extend the research in two directions. First, more information is needed on the types of health problems related to young adults becoming members of Cluster 3. To what extent does membership in Cluster 3 relate to mental health and disabilities, which make it difficult, if not impossible, to attain an education and transition to the labour market? [15] Second, the analyses should be extended to examine the associations between ACEs and the trajectories prevalent in Cluster 2. As seen from our study, many of the ACEs, most notably parental divorce and witnessing violence, increased the likelihood of being a member of Cluster 2, where the mean duration outside the labour market was also higher than that in Cluster 1. It remains to be seen to what extent this association is related to young adults’ transition into their own family of destination (e.g., parental leave due to becoming a parent earlier), or with the type of education more prevalent in this cluster and a related higher risk of sick leave and unemployment.

Future research focusing on better operationalisation and collection of data related to the mechanisms causing labour market trajectories with very little labour market participation would be beneficial for enhancing the discussion of which policies could potentially alleviate the harmful effects of ACEs. Another area of research that could be relevant for the discussion of the association between ACEs and labour market trajectories is the possible moderating effect of context, which could include individual and family resources, as well as support from extended family, the school context (teachers or classmates), the neighbourhood, or access to government educational and/or employment programmes.

Research on the negative effects of ACEs points to the relevance of contextual resources [53, 54]. Household SEP in childhood, as well as ACEs, are associated determinants of later labour market trajectories [16, 17]. We did not look at this association in our study, but we included household SEP in our analyses. Pitkänen et al. [16] and Schurer et al. [17] discussed whether parental SEP or ACEs were most important for educational and employment transitions, and their results diverged. In our study, ACEs were associated with a higher probability of being a member of Cluster 2, which included some educational experience. This suggests that it would be beneficial to research more specific associations between ACEs and transitions from one part of the educational system to another.

Accounting for potential variations in the effects of different combinations of ACEs [7] and for variations in age when experiencing them could also be important [19]. As mentioned in the introduction, one could hypothesise that experiencing, for example, parental death or unemployment in adolescence might be a particularly vulnerable time when it comes to subsequent labour market trajectories because at this chronological age, important processes related to decisions about education and future work are formed. Future studies that examine the timing of ACEs are needed for this reason, preferably using prospective and objective register measures of ACEs instead of retrospective self-reported ones. The study should be extended to different contexts, preferably examining the extent to which the association between ACEs and labour market trajectories on the margins of the labour market can be replicated in other welfare state regimes. It may be that ACEs in other countries with less extensive provisions for people outside the labour market would lead to more labour market participation for those experiencing ACEs, although they might experience lower incomes or higher levels of poverty compared to those without exposure to ACEs.

## Conclusions

The main finding of this study is that there might still be a need for adolescents and young adults to draw on resources from social workers or health professionals to circumvent the possible negative consequences of certain adverse childhood experiences. This is most obviously the case when it comes to being abused and witnessing violent events. However, this is also the case for more prevalent experiences, such as parental drug or alcohol abuse and parental divorce; the latter was experienced by nearly one-fifth of the birth cohort in question and by a higher number in more urban populations. This study suggests that even if these experiences occur in childhood, their consequences are of a more lasting duration. Therefore, policies identifying high-risk young adults and helping them through life crises related to ACEs might be one way to reduce one possible pathway transmitting social inequalities from one generation to the next.

## Supplementary Information

**Additional file 1: Table S1.** Sociodemographic characteristics by clusters of labour market trajectories. (Percent) Supplementary table using complete case analysis. **Table S2**. Adverse childhood experiences (ACEs) (until age 16) and labour market trajectories (age 16–32). Multinomial logistic regression (95% CI). Supplementary table using complete case analysis.

## Data Availability

As the study included sensitive information covered by GDPR, restrictions apply to the availability of data that is not publicly available. Access to data is only possible after reasonable request to the authors and only through Statistics Denmark. Contact should be taken to Claus. D. Hansen (clausdh@socsci.aau.dk).

## References

[1] Edwards R, Gillies V, White S (2019). Introduction: adverse childhood experiences (ACES) – implications and challenges. Soc Policy Soc.

[2] Monnat SM, Chandler RF (2015). Long-term physical health consequences of adverse childhood experiences. Sociol Q.

[3] Chapman DP, Whitfield CL, Felitti VJ, Dube SR, Edwards VJ, Anda RF (2004). Adverse childhood experiences and the risk of depressive disorders in adulthood. J Affect Disord.

[4] Danese A, Moffitt TE, Harrington H, Milne BJ, Polanczyk G, Pariante CM, Poulton R, Caspi A (2009). Adverse childhood experiences and adult risk factors for age-related disease: depression, inflammation, and clustering of metabolic risk markers. Arch Pediatr Adolesc Med.

[5] Hughes K, Bellis MA, Hardcastle KA, Sethi D, Butchart A, Mikton C, Jones L, Dunne MP (2017). The effect of multiple adverse childhood experiences on health: a systematic review and meta-analysis. Lancet Public Health.

[6] Hughes K, Bellis MA, Sethi D, Andrew R, Yon Y, Wood S, Ford K, Baban A, Boderscova L, Kachaeva M, Makaruk K, Markovic M, Povilaitis R, Raleva M, Terzic N, Veleminsky M, Włodarczyk J, Zakhozha V (2019). Adverse childhood experiences, childhood relationships and associated substance use and mental health in young Europeans. Eur J Pub Health.

[7] Brown SM, Rienks S, McCrae JS, Watamura SE (2019). The co-occurrence of adverse childhood experiences among children investigated for child maltreatment: a latent class analysis. Child Abuse Negl.

[8] Lee RD, Chen J (2017). Adverse childhood experiences, mental health, and excessive alcohol use: examination of race/ethnicity and sex differences. Child Abuse Negl.

[9] Meadows AL, Strickland JC, Kerr MS, Rayapati AO, Rush CR (2019). Adverse childhood experiences, tobacco use, and obesity: a crowdsourcing study. Subst Use Misuse.

[10] Björkenstam E, Kosidou K, Björkenstam C (2016). Childhood household dysfunction and risk of self-harm: a cohort study of 107 518 young adults in Stockholm County. Int J Epidemiol.

[11] Frankenberger DJ, Clements-Nolle K, Yang W (2015). The association between adverse childhood experiences and alcohol use during pregnancy in a representative sample of adult women. Womens Health Issues.

[12] Liu Y, Croft JB, Chapman DP, Perry GS, Greenlund KJ, Zhao G, Edwards VJ (2013). Relationship between adverse childhood experiences and unemployment among adults from five US states. Soc Psychiatry Psychiatr Epidemiol.

[13] Lund T, Andersen JH, Winding TN, Biering K, Labriola M (2013). Negative life events in childhood as risk indicators of labour market participation in young adulthood: a prospective birth cohort study. PLoS One.

[14] Metzler M, Merrick MT, Klevens J, Ports KA, Ford DC (2017). Adverse childhood experiences and life opportunities: shifting the narrative. Child Youth Serv Rev.

[15] Björkenstam E, Hjern A, Vinnerljung B (2017). Adverse childhood experiences and disability pension in early midlife: results from a Swedish national cohort study. Eur J Pub Health.

[16] Pitkänen J, Remes H, Moustgaard H, Martikainen P (2019). Parental socioeconomic resources and adverse childhood experiences as predictors of not in education, employment, or training: a Finnish register-based longitudinal study. J Youth Stud.

[17] No IZADP, Schurer S, Trajkovski K, Schurer S, Trajkovski K (2018). Discussion paper series understanding the mechanisms through which adverse childhood experiences affect lifetime economic outcomes.

[18] Hardcastle K, Bellis MA, Ford K, Hughes K, Garner J, Ramos RG (2018). Measuring the relationships between adverse childhood experiences and educational and employment success in England and Wales: findings from a retrospective study. Public Health.

[19] Flaherty EG, Thompson R, Dubowitz H, Harvey EM, English DJ, Proctor LJ, Runyan DK (2013). Adverse childhood experiences and child health in early adolescence. JAMA Pediatr.

[20] Walsh D, McCartney G, Smith M, Armour G (2019). Relationship between childhood socioeconomic position and adverse childhood experiences (ACEs): a systematic review. J Epidemiol Community Health.

[21] Wiborg ØN, Møberg RJ (2010). Social origin and the risks of disadvantage in Denmark and Norway: the early life course of young adults. Work Employ Soc.

[22] Thern E, de Munter J, Hemmingsson T, Rasmussen F (2017). Long-term effects of youth unemployment on mental health: does an economic crisis make a difference?. J Epidemiol Community Health.

[23] Virtanen P, Lintonen T, Westerlund H, Nummi T, Janlert U, Hammarström A (2016). Unemployment in teens and trajectories of alcohol consumption in adulthood. BMJ Open.

[24] Schmillen A, Umkehrer M (2017). The scars of youth: effects of early-career unemployment on future unemployment experience. Int Labour Rev.

[25] Piccinelli M, Wilkinson G (2000). Gender differences in depression: critical review. Br J Psychiatry.

[26] Haatainen KM, Tanskanen A, Kylmä J, Honkalampi K, Koivumaa-Honkanen H, Hintikka J, Antikainen R, Viinamäki H (2003). Gender differences in the association of adult hopelessness with adverse childhood experiences. Soc Psychiatry Psychiatr Epidemiol.

[27] Gallo EAG, Munhoz TN (2018). Loret de Mola C, Murray J. gender differences in the effects of childhood maltreatment on adult depression and anxiety: a systematic review and meta-analysis. Child Abuse Negl.

[28] Directorate-General for Employment SA and I European C, Verashchagina A, Bettio F. Gender segregation in the labour market: Root causes, implications and policy responses in the EU. LU: Publications Office of the European Union; 2009. https://data.europa.eu/doi/10.2767/1063. Accessed 11 Aug 2021.

[29] Carlson JS, Yohannan J, Darr CL, Turley MR, Larez NA, Perfect MM (2020). Prevalence of adverse childhood experiences in school-aged youth: a systematic review (1990–2015). Int J Sch Educ Psychol.

[30] Borchorst A, Siim B (2002). The women-friendly welfare states revisited. NORA—Nordic J Fem Gender Res.

[31] Ploug N (2012). The Nordic child care regime—history, development and challenges. Child Youth Serv Rev.

[32] Hestbæk A-D (2011). Denmark: A child welfare system under reframing.

[33] Petersson F, Baadsgaard M, Thygesen LC (2011). Danish registers on personal labour market affiliation. Scand J Public Health.

[34] Jensen VM, Rasmussen AW (2011). The Danish education registers. Scand J Public Health.

[35] Hjollund NH, Larsen FB, Andersen JH (2007). Register-based follow-up of social benefits and other transfer payments: accuracy and degree of completeness in a Danish interdepartmental administrative database compared with a population-based survey. Scand J Public Health.

[36] Pedersen CB (2011). The Danish civil registration system. Scand J Public Health.

[37] Murtagh F, Legendre P (2014). Ward’s hierarchical agglomerative clustering method: which algorithms implement Ward’s criterion?. J Classif.

[38] Abbott A, Tsay A (2000). Sequence analysis and optimal matching methods in sociology. Sociol Methods Res.

[39] Gabadinho A, Ritschard G, Müller NS, Studer M. Analyzing and visualizing state sequences in R with TraMineR. J Stat Softw. 2011;40(4). 10.18637/jss.v040.i04.

[40] Studer M, Ritschard G (2016). What matters in differences between life trajectories: A comparative review of sequence dissimilarity measures. J Royal Stat Soc.

[41] Hubert LJ, Levin JR (1976). A general statistical framework for assessing categorical clustering in free recall. Psychol Bull.

[42] Hubert L, Arabie P (1985). Comparing partitions. J Classif.

[43] Studer M. WeightedCluster library manual: A practical guide to creating typologies of trajectories in the social sciences with R. Lausanne; 10.12682/lives.2296-1658.2013.24. Retrieved from https://archive-ouverte.unige.ch/unige:78576.

[44] Hennig C, Liao TF (2010). Comparing latent class and dissimilarity-based clustering for mixed type variables with application to social stratification.

[45] Hardt J, Rutter M (2004). Validity of adult retrospective reports of adverse childhood experiences: review of the evidence. J Child Psychol Psychiatry.

[46] Hardt J, Vellaisamy P, Schoon I (2010). Sequelae of prospective versus retrospective reports of adverse childhood experiences. Psychol Rep.

[47] Newcomb MD, Huba GJ, Bentler PM (1981). A multidimensional assessment of stressful life events among adolescents: derivation and correlates. J Health Soc Behav.

[48] Turner RJ, Wheaton B, Lloyd DA (1995). The epidemiology of social stress. Am Sociol Rev.

[49] Bethell CD, Carle A, Hudziak J, Gombojav N, Powers K, Wade R, Braveman P (2017). Methods to assess adverse childhood experiences of children and families: toward approaches to promote child well-being in policy and practice. Acad Pediatr.

[50] Stata Statistical Software: Release 16. College Station, TX: StataCorp; 2019. https://www.stata.com/support/faqs/resources/citing-software-documentation-faqs/.

[51] Gabadinho A, Studer M, Müller N, Bürgin R, Fonta P-A, Ritschard G (2020). Trajectory miner: a toolbox for exploring and rendering sequences.

[52] Strompolis M, Tucker W, Crouch E, Radcliff E (2019). The intersectionality of adverse childhood experiences, race/ethnicity, and income: implications for policy. J Prev Interv Community.

[53] Nurius PS, Logan-Greene P, Green S (2012). Adverse childhood experiences (ACE) within a social disadvantage framework: distinguishing unique, cumulative, and moderated contributions to adult mental health. J Prev Interv Community..

[54] Logan-Greene P, Green S, Nurius PS, Longhi D (2014). Distinct contributions of adverse childhood experiences and resilience resources: a cohort analysis of adult physical and mental health. Soc Work Health Care.

