# Supplementary material for: The importance of adverse childhood experiences for labour market trajectories over the life course: a longitudinal study
Source: BMC Public Health. 2021 Nov 8;21:2044. doi: 10.1186/s12889-021-12060-5 (PMC8577013; doi:10.1186/s12889-021-12060-5)
Supplement: Supplementary file 1 — Additional file 1: Table S1. Sociodemographic characteristics by clusters of labour market trajectories. (Percent) Supplementary table using complete case analysis. Table S2. Adverse childhood experiences (ACEs) (until age 16) and labour market trajectories (age 16–32). Multinomial logistic regression (95% CI). Supplementary table using complete case analysis. [file 12889_2021_12060_MOESM1_ESM.docx]

**Table S1. Sociodemographic characteristics by clusters of labour market trajectories. (Percent) Supplementary table using complete case analysis.**

|  | **N** | **Grand mean** | **Cluster 1** | **Cluster 2** | **Cluster 3** |
| --- | --- | --- | --- | --- | --- |
| **Sociodemographic characteristics and class of origin** |  |  |  |  |  |
| Gender (% female) | 3100 | 48.0 | 60.0 | 38.6 | 50.0 |
| Household disposable income (mean equivalented in Dkr) | 3056 | 106,944 | 113,386 | 103,807 | 86,571 |
| Parental education (% unskilled) | 3052 | 38.6 | 26.4 | 46.6 | 56.1 |
| Lone parent (% living with single parent) | 3079 | 8.5 | 7.0 | 9.0 | 15.4 |
| Number of children in household (mean) | 3101 | 2.4 | 2.5 | 2.4 | 2.1 |
| **Mean duration of state spaces (in months)** |  |  |  |  |  |
| Basic school |  | 15.5 | 15.5 | 15.4 | 16.6 |
| Upper secondary |  | 19.5 | 33.7 | 11.0 | 2.6 |
| Vocational training |  | 20.8 | 4.1 | 33.5 | 13.3 |
| Higher education |  | 27.5 | 60.7 | 6.1 | 0.6 |
| Other education |  | 2.9 | 2.0 | 3.4 | 4.4 |
| Employed |  | 84.8 | 63.8 | 105.9 | 17.1 |
| Outside labour market |  | 33.0 | 24.3 | 28.7 | 149.4 |
| N (for register-based measures) (%) |  | 3264 | 1297  (39.7) | 1797  (55.1) | 170  (5.2) |
| **Adverse childhood experiences** |  |  |  |  |  |
| Mean number of adverse childhood experiences | 1788 | 0.7 | 0.6 | 0.8 | 1.5 |
| Parental divorce |  | 18.8 | 14.5 | 21.9 | 35.0 |
| Parental unemployment |  | 16.8 | 13.5 | 18.8 | 30.5 |
| Parental death |  | 4.3 | 3.4 | 4.8 | 11.7 |
| Parental drug or alcohol abuse |  | 11.6 | 9.0 | 13.2 | 22.0 |
| Abuse or neglect |  | 4.6 | 3.3 | 4.9 | 19.0 |
| Witness violent event |  | 4.8 | 2.7 | 3.1 | 10.0 |
| Parental accident or serious illness |  | 12.2 | 11.2 | 12.3 | 20.3 |
|  |  |  |  |  |  |

**Table S2. Adverse childhood experiences (ACEs) (until age 16) and labour market trajectories (age 16–32). Multinomial logistic regression (95 % CI). Supplementary table using complete case analysis.**

|  | **Cluster membership** | **Model 1 (Bivariate associations)** | **Model 2 (Adjusted for SES)** |
| --- | --- | --- | --- |
| **Adverse childhood experiences  (N = 1,710)** |  |  |  |
| No. of adverse childhood experiences | 1 | 1 (ref) | 1 (ref) |
|  | 2 | 1.22 (1.11-1.35) | 1.17 (1.05-1.31) |
|  | 3 | 1.77 (1.45-2.18) | 1.67 (1.34-2.10) |
| Parental divorce | 1 | 1 (ref) | 1 (ref) |
|  | 2 | 1.64 (1.27-2.12) | 1.63 (1.21-2.20) |
|  | 3 | 2.70 (1.43-5.09) | 2.09 (0.99-4.41) |
| Parental death | 1 | 1 (ref) | 1 (ref) |
|  | 2 | 1.12 (0.67-1.88) | 0.97 (0.55-1.69) |
|  | 3 | 3.19 (1.18-8.63) | 2.39 (0.82-7.00) |
| Parental drug or alcohol abuse | 1 | 1 (ref) | 1 (ref) |
|  | 2 | 1.46 (1.06-1.99) | 1.33 (0.96-1.85) |
|  | 3 | 2.87 (1.41-5.84) | 2.30 (1.10-4.81) |
| Parental unemployment | 1 | 1 (ref) | 1 (ref) |
|  | 2 | 1.45 (1.11-1.89) | 1.20 (0.91-1.60) |
|  | 3 | 2.60 (1.36-4.98) | 1.99 (1.01-3.94) |
| Abuse or neglect | 1 | 1 (ref) | 1 (ref) |
|  | 2 | 1.24 (0.74-2.08) | 1.34 (0.79-2.28) |
|  | 3 | 6.53 (2.89-14.74) | 6.00 (2.59-13.92) |
| Witness violent event | 1 | 1 (ref) | 1 (ref) |
|  | 2 | 2.08 (1.26-3.45) | 1.87 (1.11-3.16) |
|  | 3 | 3.03 (1.01-9.11) | 2.60 (0.85-7.98) |
| Parental accident or serious illness | 1 | 1 (ref) | 1 (ref) |
|  | 2 | 1.05 (0.77-1.42) | 0.98 (0.72-1.35) |
|  | 3 | 2.28 (1.13-4.59) | 2.00 (0.98-4.10) |
| **Sociodemographic characteristics and class of origin (N = 2,996)** |  |  |  |
| Gender (male vs. female) | 1 | 1 (ref) | 1 (ref) |
|  | 2 | 0.41 (0.36-0.48) | 0.40 (0.35-0.47) |
|  | 3 | 0.67 (0.47-0.96) | 0.64 (0.45-0.92) |
| Parents unskilled vs. Skilled | 1 | 1 (ref) | 1 (ref) |
|  | 2 | 2.42 (2.06-2.84) | 2.39 (2.03-2.82) |
|  | 3 | 3.69 (2.56-5.30) | 3.46 (2.40-4.99) |
| Household disposable income (in 100.000 Dkr) | 1 | 1 (ref) | 1 (ref) |
|  | 2 | 0.75 (0.65-0.87) | 0.77 (0.65-0.90) |
|  | 3 | 0.40 (0.26-0.63) | 0.43 (0.27-0.70) |
| Lone parent vs. two parents | 1 | 1 (ref) | 1 (ref) |
|  | 2 | 1.31 (0.98-1.74) | 1.20 (0.89-1.63) |
|  | 3 | 2.51 (1.48-4.25) | 2.01 (1.16-3.49) |
| Number of children in household | 1 | 1 (ref) | 1 (ref) |
|  | 2 | 0.94 (0.88-1.01) | 0.93 (0.87-1.01) |
|  | 3 | 0.88 (0.73-1.07) | 0.86 (0.71-1.06) |
